# Efficacy of traditional Chinese medicine in improving nutritional status among Chinese maintenance hemodialysis patients: a meta-analysis of the last decade

**DOI:** 10.3389/fnut.2026.1852062

**Published:** 2026-08-13

**Authors:** Buqing Xu, Xue Cao

**Affiliations:** Blood Purification Center, Zhejiang Hospital, Hangzhou, Zhejiang, China

**Keywords:** maintenance hemodialysis, malnutrition, meta-analysis, traditional Chinese medicine, uremic

## Abstract

**Background:**

Malnutrition is a common and severe complication in maintenance hemodialysis patients, which significantly increases the risk of adverse clinical outcomes. Traditional Chinese medicine (TCM) has emerged as a promising adjunctive therapy for nutritional improvement, yet its efficacy and underlying mechanisms require systematic clarification. This study synthesized the evidence to explore the role of TCM in managing malnutrition in hemodialysis populations. These findings aim to inform evidence-based decisions regarding its clinical applicability.

**Method:**

A systematic literature search was conducted across four international biomedical databases (e.g., PubMed, Embase, Web of Science, ClinicalTrials.gov) and three Chinese scholarly platforms (CNKI, Wanfang, CQVIP), with the search period spanning from March 2016 to March 2026. We systematically reviewed randomized controlled trials (RCTs) evaluating TCM interventions on nutritional status improvement in patients undergoing maintenance hemodialysis. Stata version 12.0 statistical software (StataCorp LP, College Station, Texas) was adopted as statistical software. When I^2^ < 50%, we consider the data has no obvious heterogeneity, and we conduct a meta-analysis using the fixed-effect model. Otherwise, the random-effect model was performed.

**Results:**

This meta-analysis included 26 randomized controlled trials (RCTs) conducted in China, involving a total of 2,263 patients with uremia. Among them, 1,135 patients received traditional Chinese medicine treatment in addition to maintenance hemodialysis and standard care, while the remaining 1,128 patients did not receive TCM treatment. Our analysis found that traditional Chinese medicine significantly improved overall response rate compared with the control group (RR = 1.460; 95% CIs: 1.310 to 1.626; *p* < 0.001). For nutritional indicators, TCM was associated with significant improvements in serum albumin (WMD = 3.009; 95% CIs: 2.149 to 3.869; *p* < 0.001), hemoglobin (WMD = 7.975; 95% CIs: 5.901 to 10.048; *p* < 0.001), triceps skinfold thickness (WMD = 1.119; 95% CIs: 0.605 to 1.634; *p* < 0.001), residual kidney function (WMD = 0.991; 95% CIs: 0.685 to 1.297; *p* < 0.001), transferrin (WMD = 0.607; 95% CIs: 0.266 to 0.948, *p* < 0.001), total cholesterol (WMD = 0.467; 95% CIs: 0.175 to 0.759; *p* = 0.002), and modified quantitative subjective global assessment scores (WMD = −2.613; 95% CIs: −3.694 to −1.532; *p* < 0.001). No significant differences were observed in body mass index, mid-arm circumference, leptin, subjective global assessment, or malnutrition inflammation score. Four studies reported adverse events, with no significant difference between groups (RR = 1.335; 95% CIs: 0.358 to 4.972; *p* = 0.667).

**Conclusion:**

Among the included patients undergoing maintenance hemodialysis, traditional Chinese medicine demonstrated efficacy in improving nutritional status, with significant improvements observed across multiple nutritional indicators and no increase in the risk of adverse events. Nevertheless, given the inherent heterogeneity among the included studies, further high-quality evidence is warranted to support these findings.

## Introduction

Currently, chronic kidney disease (CKD) has become one of the global public health challenges. End-stage renal disease (ESRD) represents the terminal stage of CKD and is associated with high morbidity and mortality (1, 2). In 2023, the global number of cases of kidney failure requiring replacement therapy (KFRT) was 4.59 million (3). Over the past three decades, the prevalence of KFRT has steadily increased worldwide. Maintenance hemodialysis (MHD) is the most common and primary form of renal replacement therapy (RRT) for patients with ESRD (4). However, as a form of renal replacement therapy, MHD cannot completely eliminate the toxins accumulated in the body of patients with end-stage renal disease. Moreover, it may lead to the loss of various nutrients, affect the process of protein synthesis, and accelerate protein catabolism, thereby contributing to the development of malnutrition in dialysis patients (5). According to surveys, the incidence of malnutrition in patients undergoing MHD ranges from 28 to 54% (6, 7). Malnutrition not only affects patients’ quality of life, it also increases the risk of complications such as cardiovascular disease and infection, raises the risk of hospitalization, and may even lead to death (8). Therefore, improving malnutrition in patients undergoing MHD is of great significance.

Currently, a well-established system is in place for assessing, managing, and evaluating the nutritional status of patients undergoing maintenance hemodialysis, including dynamic monitoring of relevant indicators, dietary guidance, and the use of nutritional supplements such as ketoanalogues and oral amino acids (9). However, with prolonged dialysis duration, patients often develop issues such as reduced appetite, toxin accumulation, and microinflammation, for which modern medicine lacks effective and feasible interventions (5). Recent studies suggest, Traditional Chinese medicine (TCM) has demonstrated significant benefits in improving nutritional status in patients undergoing hemodialysis (10). For example, the study by Xingyue Quan et al. (11) concluded that the combination of Heidihuang Powder and rHuEPO significantly improved anemia and malnutrition in patients undergoing maintenance hemodialysis, with a favorable safety profile (11). Kejia Li stated that adding Jianpi Huazhuo Decoction to conventional Western medicine treatment can alleviate malnutrition symptoms in patients undergoing maintenance hemodialysis, improve blood biochemical parameters and anthropometric indicators, and enhance overall nutritional status (12). Additionally, Jiebin Chen, Xiongliang Huang, Zuhong Zhang, et al. also conducted relevant studies (13–21). Nevertheless, the efficacy and limitations of TCM in addressing malnutrition in patients undergoing maintenance hemodialysis remain unclear. This study employed a meta-analysis to systematically evaluate the research status of TCM interventions for malnutrition in patients undergoing MHD over the past decade, aiming to provide insights and establish a foundation for developing clinical TCM treatment strategies for MHD-related malnutrition.

## Materials and methods

This meta-analysis was not registered in a public registry. The PRISMA guidelines were followed throughout the conduct of this systematic review and meta-analysis.

### Literature search strategy

**Figure 1:**
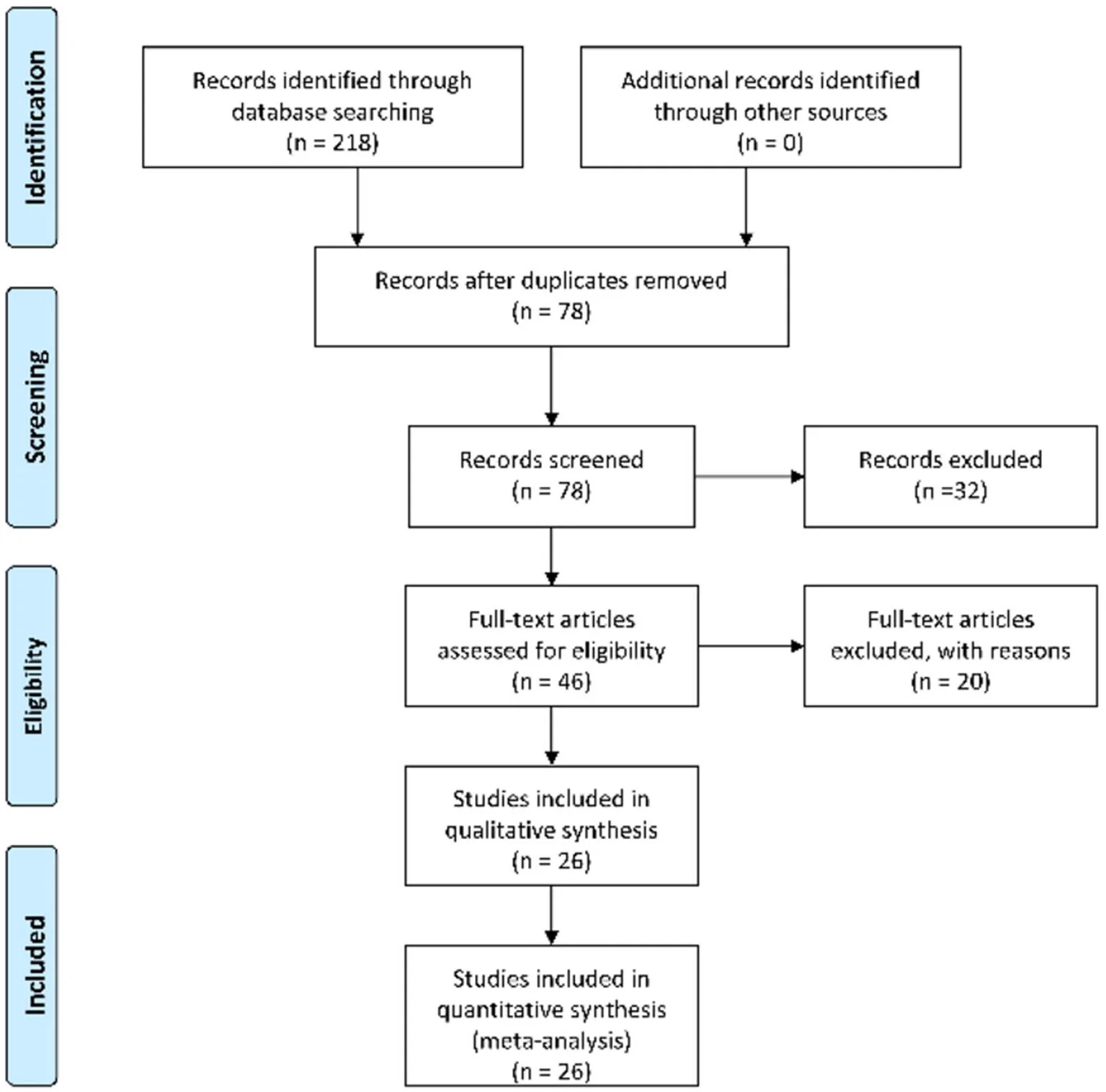
The depicted screening study process.

A systematic literature search was performed in multiple electronic databases, including PubMed, Embase, Web of Science, ClinicalTrials.gov, and three major Chinese scholarly platforms: CNKI, Wanfang, and CQVIP. The search covered the period from March 2016 to March 2026. We conducted a systematic review of randomized controlled trials to assess the therapeutic effects of traditional Chinese medicine on nutritional status in patients receiving maintenance hemodialysis. The search strategy incorporated both free-text terms and MeSH (Medical Subject Headings) terms related to maintenance hemodialysis, malnutrition, traditional Chinese medicine, and meta-analysis. A manual search was additionally conducted to identify eligible randomized controlled trials. The search was conducted with language restrictions to Chinese and English. The complete search strings for each database are provided in Supplementary file. The full detail of the search protocol is outlined in Figure 1.

### Inclusion and exclusion criteria

Studies were incorporated into the analysis according to the following criteria in line with the PICOS framework: (I) Participants: Patients undergoing maintenance hemodialysis. (II) Interventions: Patients with uremia received traditional Chinese medicine (TCM) interventions, including but not limited to Chinese herbal medicine (various formulations), Chinese patent medicines, herbal injections (intravenous or acupoint injection), acupuncture, and auricular therapy. All patients underwent maintenance hemodialysis and received treatment for underlying diseases, with or without concurrent conventional Western medicine. (III) Comparisons: Comparisons were made between maintenance hemodialysis patients who received traditional Chinese medicine (TCM) interventions and those who did not, in terms of improvement in nutritional status. (IV) Outcome measures: studies needed to report one or more of the following endpoints: albumin (Alb), hemoglobin (HGB), body mass index (BMI), triceps skinfold thickness (TSF), mid-arm circumference (MAC), leptin, residual kidney function (RKF), transferrin (TF), total cholesterol (TC/CHO), Subjective Global Assessment (SGA), Modified Quantitative Subjective Global Assessment (MQSGA), Malnutrition-Inflammation Score (MIS), and total effective rate. (V) Study design: Eligible studies were restricted to full-text randomized controlled trials (RCTs) with complete data reporting.

Studies were excluded if they (I) did not meet the predefined inclusion criteria; (II) were non-randomized controlled trial designs (e.g., editorials, conference proceedings, commentaries, reviews, clinical conference reports, or abstracts); (III) involved animal subjects; (IV) employed inconsistent or heterogeneous interventions; or (V) were duplicate publications from the same database.

### Data extraction and outcome measures

Two reviewers independently selected studies and extracted data. Disagreements were resolved through discussion or by consulting a third author. Extracted data were categorized into four domains: (1) overall efficacy outcome, defined as the overall response rate; (2) laboratory nutritional parameters, including albumin (Alb), hemoglobin (HGB), leptin, residual kidney function (RKF), transferrin (TF), and total cholesterol (TC/CHO); (3) anthropometric measurements, comprising body mass index (BMI), triceps skinfold thickness (TSF), and mid-arm circumference (MAC); and (4) nutritional assessment scales, namely Subjective Global Assessment (SGA), Modified Quantitative Subjective Global Assessment (MQSGA), and Malnutrition-Inflammation Score (MIS).

### Risk of bias assessment

Two independent authors assessed the risk of bias of the included randomized controlled trials (RCTs) using the Cochrane Collaboration’s Risk of Bias tool. The assessment encompassed the following domains: sequence generation, allocation concealment, blinding of outcome assessors, incomplete outcome data, selective outcome reporting, and other potential sources of bias. Two reviewers independently assessed bias risk (high/unclear/low), with disagreements resolved by consensus or third reviewer involvement. Most RCTs demonstrated moderate quality. Detailed results of the quality assessment are presented in Table 1.

**Table 1:** Cochrane collaboration’s tool for quality assessment in all included trials.

Trials (authors)	Year	Sequence generation	Allocation concealment	Blinding of outcome assessors	Incomplete outcome data	Selective outcome reporting	Others
Ren et al. (19)	2016	Low	Unclear	Low	Low	Low	Low
Xu et al. (17)	2016	Unclear	Low	Low	Low	Unclear	Unclear
Yang et al. (18)	2016	Low	Unclear	Low	Low	Low	Unclear
Yu et al. (16)	2017	Unclear	Low	Low	Low	Low	Low
Sun et al. (22)	2017	Low	Unclear	Low	Low	Low	Low
Chen et al. (23)	2018	Low	Low	Unclear	Low	Low	Unclear
Chen et al. (15)	2018	Unclear	Low	Low	Low	Low	Low
Li et al. (24)	2018	Low	Unclear	Low	Low	Low	Unclear
Yuan et al. (25)	2018	Unclear	Low	Low	Low	Low	Low
Huang et al. (13)	2019	Low	Unclear	Low	Low	Low	Low
Li et al. (26)	2019	Low	Low	Unclear	Low	Low	Unclear
Li et al. (12)	2020	Low	Unclear	Low	Low	Low	Unclear
Sun et al. (27)	2020	Unclear	Low	Low	Low	Low	Low
Wang et al. (28)	2021	Low	Unclear	Low	Low	Low	Low
Yan et al. (29)	2021	Low	Low	Unclear	Low	Low	Unclear
Yan et al. (30)	2021	Unclear	Low	Low	Low	Low	Low
Zhang et al. (14)	2021	Low	Unclear	Low	Low	Low	Low
Liu et al. (32)	2021	Low	Low	Unclear	Low	Low	Unclear
Hao et al. (33)	2021	Low	Unclear	Low	Low	Low	Low
Wen et al. (34)	2022	Unclear	Low	Low	Low	Unclear	Unclear
Xu et al. (35)	2022	Low	Unclear	Low	Low	Low	Unclear
Chen et al. (21)	2023	Unclear	Low	Low	Low	Low	Low
Zhang et al. (20)	2023	Low	Unclear	Low	Low	Low	Low
Quan et al. (11)	2024	Low	Low	Unclear	Low	Low	Unclear
Tang et al. (36)	2022	Unclear	Low	Low	Low	Low	Low
Zhang et al. (14)	2021	Unclear	Low	Low	Low	Low	Low

### Statistical analysis

Meta-analyses were performed with Stata version 12.0. Assessing heterogeneity with I^2^ statistic (<50% threshold). Random-effects meta-analysis followed Cochrane guidelines. For continuous outcomes, Alb, HGB, BMI, TSF, MAC, leptin, RKF, TF, TC/CHO, SGA, MQSGA, MIS, we reported continuous data using weighted mean differences (WMD) and 95% confidence intervals (CIs). For categorical variables, including overall effectiveness rate and adverse events, we calculated risk ratios (RR) with 95% CIs. Statistical significance was set at *p* < 0.05.

## Results

### Search results

As shown in Figure 1, the literature screening was conducted in accordance with predefined inclusion and exclusion criteria. A total of 218 records were initially identified through database searches. After removing 140 duplicates using Endnote and manual verification, 78 unique articles were screened based on titles and abstracts, resulting in the exclusion of 32 records that did not meet the eligibility criteria. The remaining 46 full-text articles were assessed in detail; of these, 20 were excluded for specific reasons. Ultimately, 26 randomized controlled trials (RCTs) satisfied all inclusion criteria and were included in the meta-analysis (11–36).

### Study characteristics and quality assessment

Table 2 summarizes the baseline characteristics and demographic data of 2,263 patients with uremia enrolled in 26 trials conducted in China. All 26 included studies were randomized controlled trials (RCTs). Among the 2,263 patients with uremia, 1,135 received TCM treatment in addition to hemodialysis and basic care, while the remaining 1,128 did not receive TCM treatment. Of these studies, 11 studies (11–16, 18–23, 25–30, 33–35) reported the dialysis vintage of patients with uremia, while 5 studies (17, 24, 31, 32, 36) did not mention it. The study duration ranged from 1 to 6 treatment periods, and 5 studies provided information on adverse reactions (18–20, 27, 28). Among them, the cases that dropped out or withdrew from the study due to adverse reactions were Yang et al. (18) 8 cases of arteriovenous fistula occlusion, 2 cases of severe hypotension, and 3 cases of diarrhea intolerance caused by the use of Niaoduqing Granules. In the study by Ren et al. (19) the intervention was acupoint injection of TCM, and noticeable redness and swelling at the injection site were observed in two cases. In the study by Wang et al. (28) mild constipation occurred in two patients, which was alleviated after symptomatic treatment, while intermittent nausea without vomiting was reported in three patients and resolved without specific intervention. In the study by Sun et al. (27) mild oral ulcerations developed in two patients, and the symptoms were relieved after the dosage of dried ginger was reduced by half. In the study by Zhang et al. (20) one patient experienced a rash. The TCM interventions involved oral administration, external application, intravenous injection, and other routes. Among them, 19 studies focus on Chinese herbal decoctions, granules, or pastes; 4 studies investigated intravenous injections of Chinese herbal preparations; and there were 3 studies on acupoint injection of Chinese herbal medicine, 1 on acupoint application, and 1 on acupoint moxibustion. In the study by Zhang et al. (14), the TCM intervention consisted of oral administration of Shenling Baizhu Powder combined with external acupoint application of Chinese herbal medicine. In the study by Li et al. (24), oral administration of Xiangsha Yangwei Pills was combined with external moxibustion on the spleen and stomach to achieve a synergistic therapeutic effect.

**Table 2:** Baseline characteristics of the included studies.

Authors	Year	Sample size		Dialysis vintage (E/C)	Intervention		Period/ months	Adverse event	Outcomes
		Exper	Control		Exper	Control			
Ren et al. (19)	2016	30	30	19.13 ± 17.63/17.26 ± 14.46 M	Astragalus Injection (1 mL at each ST36 Zusanli point) + Hemodialysis + Underlying Disease Therapy	Hemodialysis + Conventional Therapy for Underlying Diseases.	2	Two cases of acupoint injection resulted in apparent redness and swelling.	ALB, HGB, BMI, TSF
Xu et al. (17)	2016	30	30	NA	Yi Shen Jian Pi Yang Xue Granules, Hemodialysis, and Standard Therapy for Underlying Conditions	Hemodialysis + Conventional Therapy for Underlying Diseases.	3	No adverse reactions were observed in the study subjects.	HGB, ALB, SGA
Yang et al. (18)	2016	94	92	21 (12,33) /25 (10,35) M	Niaoduqing Granules, Hemodialysis, and Standard Therapy for Underlying Conditions	Hemodialysis + Conventional Therapy for Underlying Diseases.	3	3 cases of diarrhea intolerance caused by the use of Niaoduqing Granules	ALB, HGB, MIS
Yu et al. (16)	2017	30	30	4.29 ± 1.45/4.36 ± 1.50 M	Self-Prescribed Yishen Xiezhuo Decoction, Hemodialysis, and Standard Therapy for Underlying Conditions	Hemodialysis + Conventional Therapy for Underlying Diseases.	6	NA	HGB, ALB, RKF
Sun et al. (22)	2017	37	37	5.92 ± 2.14/6.25 ± 2.08 Y	Heluo Ningfeng Granules, Hemodialysis, and Standard Therapy for Underlying Conditions	Hemodialysis + Conventional Therapy for Underlying Diseases.	2	NA	HGB, ALB,
Chen et al. (23)	2018	60	60	25.2 ± 13.6/25.6 ± 14.1 M	Shenkang Injection, Hemodialysis, and Standard Therapy for Underlying Conditions	Hemodialysis + Conventional Therapy for Underlying Diseases.	4	No adverse reactions were observed in the study subjects.	ALB, HGB, TF, BMI, TSF, AMC, MIS
Chen et al. (15)	2018	29	29	4.6 ± 2.4/5.8 ± 2.7 M	Integrated Management with the Formula for Tonifying the Spleen and Kidney, Promoting Fu-organ Function, and Resolving Turbidity, Hemodialysis and Standard Therapy for Underlying Conditions	Hemodialysis + Conventional Therapy for Underlying Diseases.	2	NA	HGB, ALB
Li et al. (24)	2018	40	40	NA	Xiangsha Yangwei Pills, Moxibustion for Spleen-Stomach Regulation, Hemodialysis, and Standard Therapy for Underlying Conditions	Hemodialysis + Conventional Therapy for Underlying Diseases.	3	NA	ALB, HGB, TF, TC/CHO, MQSGA
Yuan et al. (25)	2018	65	65	5.62 ± 1.10/5.69 ± 1.13 M	Damp-Resolving and Turbidity-Depleting Chinese Herbal Medicine, Hemodialysis, and Standard Therapy for Underlying Conditions	Hemodialysis + Conventional Therapy for Underlying Diseases.	2	NA	ALB, Leptin
Huang et al. (13)	2019	35	35	23.4 ± 9.6/22.6 ± 10.5 M	Shenkang Injection, Hemodialysis, and Standard Therapy for Underlying Conditions	Hemodialysis + Conventional Therapy for Underlying Diseases.	4	NA	ALB, HGB,
Li et al. (26)	2019	25	25	46 (29,89) /64 (30,97) M	Yiqi Jiangzhuo Therapy, Levocarnitine Supplementation, Hemodialysis, and Standard Therapy for Underlying Conditions	Levocarnitine +Hemodialysis + Conventional Therapy for Underlying Diseases.	6	No adverse reactions were observed in the study subjects.	ALB, HGB, BMI, TSF, MAC, Leptin
Li et al. (12)	2020	80	76	28.56 ± 20.43/30.75 ± 18.62 M	Jianpi Huazhuo Decoction, *α*-Keto Acid Tablets, Hemodialysis, and Standard Therapy for Underlying Conditions	α-Keto Acid Tablets+ Hemodialysis + Conventional Therapy for Underlying Diseases.	3	NA	ALB, HGB, BMI, TSF, MAC, MQSGA,
Sun et al. (27)	2020	30	30	11.14 ± 3.61/11.29 ± 3.97 M	Hei Di Huang Wan, Hemodialysis, and Standard Therapy for Underlying Conditions	Hemodialysis + Conventional Therapy for Underlying Diseases.	6	Mild oral ulcers occurred in 2 patients.	ALB, HGB, RKF
Wang et al. (28)	2021	30	30	36.80 ± 13.93/37.6 ± 13.93 M	Qizhu Capsules, Hemodialysis, and Standard Therapy for Underlying Conditions	Hemodialysis + Conventional Therapy for Underlying Diseases.	3	Mild constipation occurred in 2 cases, and occasional nausea was reported in 3 cases.	ALB, HGB
Yan et al. (29)	2021	68	68	1.25 ± 0.23/1.36 ± 0.18 Y	Shenqi Qingdu Decoction, Hemodialysis, and Standard Therapy for Underlying Conditions	Hemodialysis + Conventional Therapy for Underlying Diseases.	3	NA	ALB, HGB, RKF, TF, TC/CHO, SGA
Yan et al. (30)	2021	38	38	11.13 ± 6.85/11.05 ± 7.12 M	Shenshuai Fang, Hemodialysis, and Standard Therapy for Underlying Conditions	Hemodialysis + Conventional Therapy for Underlying Diseases.	3	NA	ALB, HGB, Leptin
Zhang et al. (31)	2021	31	32	NA	Wuse Peiyuan Guben Paste, Hemodialysis, and Standard Therapy for Underlying Conditions.	Hemodialysis + Conventional Therapy for Underlying Diseases.	2	No adverse reactions were observed in the study subjects.	ALB, HGB, SGA, TC/CHO
Liu et al. (32)	2021	30	30	NA	Runfei Yishen Decoction, Levocarnitine, Hemodialysis, and Standard Therapy for Underlying Conditions	Levocarnitine + Hemodialysis + Conventional Therapy for Underlying Diseases.	3	No adverse reactions were observed in the study subjects.	ALB, HGB
Hao et al. (33)	2021	64	64	1.42 ± 0.23/1.39 ± 0.21 Y	Jiawei Taohong Siwu Decoction, Levocarnitine Supplementation, Hemodialysis, and Standard Therapy for Underlying Conditions	Levocarnitine + Hemodialysis + Conventional Therapy for Underlying Diseases.	3	NA	ALB, HGB, RKF, TF, TC/CHO
Wen et al. (34)	2022	43	43	23.89 ± 2.15/23.86 ± 2.13 M	Shenkang Injection, Hemodialysis, and Standard Therapy for Underlying Conditions.	Hemodialysis + Conventional Therapy for Underlying Diseases.	1	NA	ALB, HGB
Xu et al. (35)	2022	82	82	25.46 ± 4.39/26.63 ± 5.81 M	Jianpi Huazhuo Paste, Hemodialysis, and Standard Therapy for Underlying Conditions.	Hemodialysis + Conventional Therapy for Underlying Diseases.	4	NA	ALB, HGB, TF, TSF, MAC, TC/CHO, MQSGA
Chen et al. (21)	2023	30	30	97.00 ± 100.53/92.97 ± 83.41 M	Acupoint Application Therapy, Hemodialysis, and Standard Therapy for Underlying Conditions.	Hemodialysis + Conventional Therapy for Underlying Diseases.	3	NA	ALB, BMI, TSF, MAC, TC/CHO, SGA
Zhang et al. (20)	2023	34	34	26.74 ± 5.08/26.67 ± 5.12 M	Acupoint Injection with Huangqi Injection, Hemodialysis, and Standard Therapy for Underlying Conditions.	Hemodialysis + Conventional Therapy for Underlying Diseases.	4	A rash occurred in 1 case.	ALB, HGB,
Quan et al. (11)	2024	41	41	16.0 ± 5.3/16.4 ± 4. 6 M	Heidi Huang San + rHuEPO+ Hemodialysis + Conventional Therapy for Underlying Diseases.	rHuEPO+ Hemodialysis + Conventional Therapy for Underlying Diseases.	2	NA	ALB, HGB, SGA
Tang et al. (36)	2022	29	27	NA	Jianpi Xiaozhi Gao + Hemodialysis + Conventional Therapy for Underlying Diseases.	Hemodialysis + Conventional Therapy for Underlying Diseases.	2	No adverse reactions were observed in the study subjects.	MQSGA, TC/CHO, BMI, TSF, MAC, HGB, ALB
Zhang et al. (14)	2021	30	30	3.8 (1–9)/3.7 (1–9) Y	Shenling Baizhu San + Traditional Chinese Medicine Acupoint Application + α-Keto Acid Tablets + Hemodialysis + Treatment of Underlying Diseases	α-Keto Acid Tablets + Hemodialysis + Treatment of Underlying Diseases	3	NA	ALB, HGB, MIS

Exper, Experiment; E/C, Experiment /Control; NA, not available; M, month; Y, year; ALB, Albumin; HGB, Hemoglobin; TC/CHO, Total Cholesterol; BMI, Body Mass Index; MAC, Mid-Upper Arm Circumference; TSF, Triceps Skinfold; TF, Transferrin; SGA, 7-piont Subjective Global Assessment; MIS, Malnutrition Inflammation Score; MQSGA, Modified quantitative subjective global assessment; RKF, Residual kidney function.

Two reviewers used the Cochrane Collaboration’s Risk of Bias tool to independently assess the methodological quality of the included randomized controlled trials (RCTs). Random sequence creation, allocation concealment, blinding of result assessors, inadequate outcome data, selective outcome reporting, and other potential biases were among the seven domains considered by the assessment. Every domain was categorized as low, high, or unclear risk, and any disagreements were settled by talking it over or consulting a third reviewer. The majority of RCTs had a methodological quality rating of moderate overall. Table 1 presents an overview of the quality assessment.

### Outcomes of meta-analysis

A total of 26 RCTs involving 2,263 patients undergoing hemodialysis for uremia were included. Detailed results are presented in Tables 3, 4, summarized as follows:

**Table 3:** The outcomes of this meta-analysis.

Outcomes	Studies numbers	Sample size		Overall effect			Heterogeneity	
		Experiment	Control	Effect estimates	95% CIs	*p*-value	I^2^(%)	*p*-value
Efficiency rate	17	577/679	380/683	RR = 1.460	1.310 to 1.626	***p* < 0.001**	54.9%	*p* = 0.003
Adverse reaction	4	8/123	6/122	RR = 1.335	0.358 to 4.972	*p* = 0.667	21.5%	*p* = 0.281
Albumin	25	1,103	1,100	WMD = 3.009	2.149 to 3.869	***p* < 0.001**	91.3%	***p* < 0.001**
Hemoglobin	25	1,098	1,095	WMD = 7.975	5.901 to 10.048	***p* < 0.001**	88.9%	***p* < 0.001**
Body Mass Index	5	196	194	WMD = 0.604	−0.352 to 1.560	*p* = 0.216	71.4%	*p* = 0.007
TSF	7	303	301	WMD = 1.119	0.605 to 1.634	***p* < 0.001**	43.1%	*p* = 0.103
MAC	6	273	271	WMD = 0.717	−0.241 to 1.675	*p* = 0.143	83.6%	***p* < 0.001**
Leptin	3	143	143	WMD = −2.012	−4.520 to 0.496	*p* = 0.116	93.7%	***p* < 0.001**
Residual kidney function	5	252	252	WMD = 0.991	0.685to 1.297	***p* < 0.001**	97.3%	***p* < 0.001**
Transferrin	5	283	283	WMD = 0.607	0.266 to 0.948	***p* < 0.001**	98.2%	***p* < 0.001**
Total Cholesterol	8	371	374	WMD = 0.467	0,175 to 0.759	*p* = 0.002	97.0%	***p* < 0.001**
SGA	5	199	199	WMD = −1.587	−5.228 to 2.053	*p* = 0.393	99.2%	***p* < 0.01**
MQSGA	4	354	350	WMD = −2.613	−3.694 to −1.532	***p* < 0.001**	81.3%	*p* = 0.001
MIS	2	94	94	WMD = −1.909	−6.111 to 2.294	*p* = 0.373	96.4%	***p* < 0.01**

CIs, confidence intervals; RR, risk ratio; WMD, weighted mean difference; Bold indicates that *p* is less than 0.05; MAC, Mid - arm Circumstance; TSF, Triceps Skinfold; SGA, 7-piont Subjective Global Assessment; MQSGA, Modified quantitative subjective global assessment; MIS, malnutrition inflammation score.

**Table 4:** Subgroup analysis of serum albumin and hemoglobin levels stratified by different treatment regimens.

Outcomes	Subgroup	Studies numbers	Sample size		Overall effect			Heterogeneity	
			Experiment	Control	Effect estimates	95% CIs	*p*-value	I^2^(%)	*p*-value
Albumin	TCM + BT vs. BT alone	20	868	868	WMD = 2.920	1.895to 3.945	***p* < 0.001**	92.5%	***p* < 0.001**
	TCM + WM + BT vs. WM + BT	5	229	225	WMD = 3.549	2.408 to 4.689	***p* < 0.001**	69.2%	*p* = 0.011
Hemoglobin	TCM + BT vs. BT alone	16	711	710	WMD = 6.825	4.560 to 9.091	***p* < 0.001**	79.7%	***p* < 0.001**
	TCM + WM + BT vs. WM + BT	9	367	365	WMD = 8.248	3.741 to 12.756	***p* < 0.001**	84.1%	***p* < 0.001**

TCM, Traditional Chinese Medicine; WM, Western medicine; BT, basic therapy. Bold values represent statistically significant results with *p* < 0.05.

### Effective rate and adverse reactions

**Figure 2:**
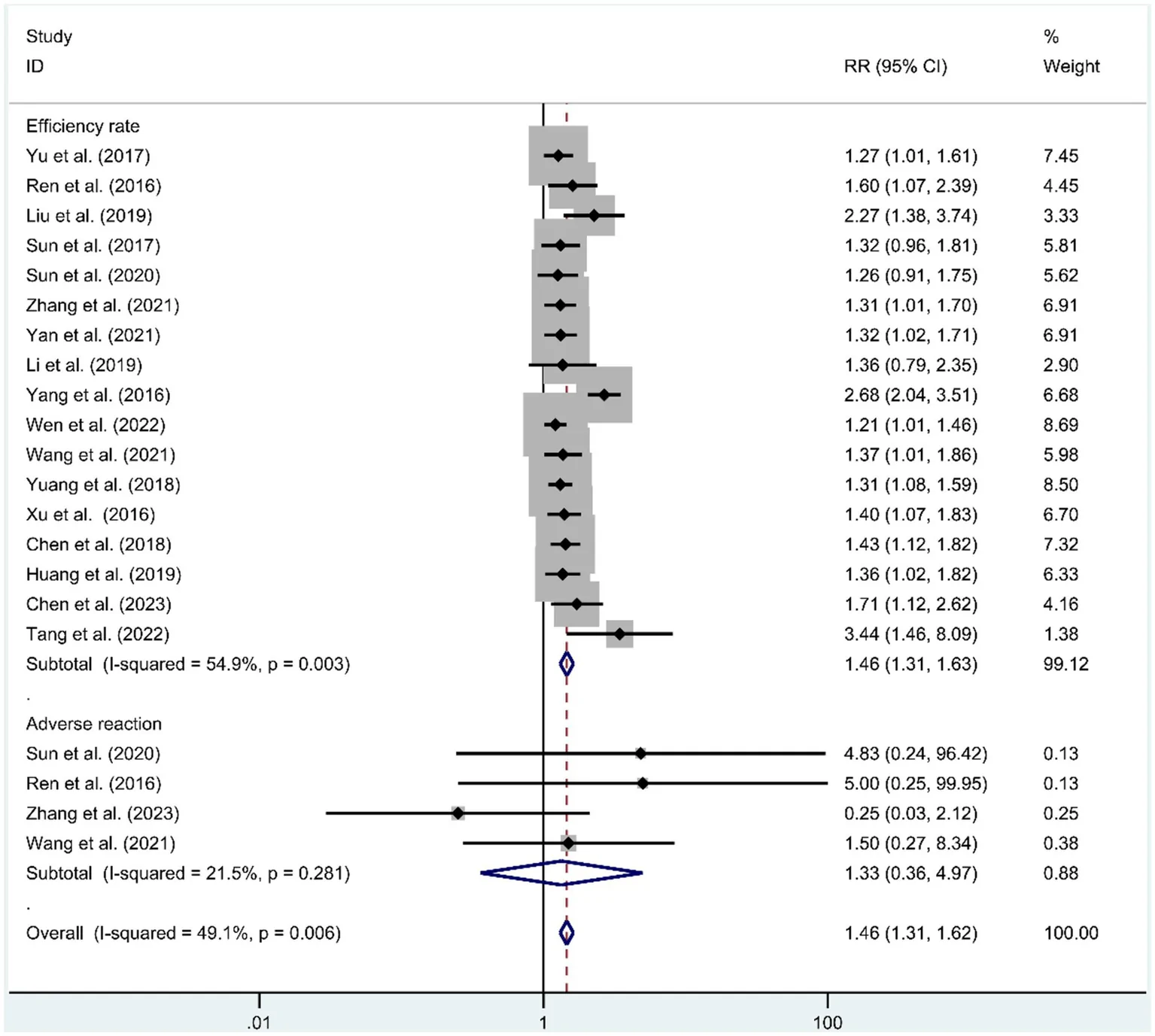
Forest plot illustrating effective rate and adverse reaction.

A total of 17 studies reported the efficiency rate. The pooled results demonstrated that the intervention group achieved a significantly higher efficiency rate compared with the control group (RR = 1.460; 95% CIs: 1.310 to 1.626; *p* < 0.001; Figure 2). Moderate heterogeneity was detected among the included studies (I^2^ = 54.9%; *p* = 0.003; Figure 2). Four studies reported data on adverse reactions. The forest plot analysis revealed no statistically significant difference between the intervention and control groups (RR = 1.335; 95% CIs: 0.358 to 4.972; *p* = 0.667; Figure 2). Low heterogeneity was detected among the studies (I^2^ = 21.5%; *p* = 0.281; Figure 2), TCM treatment improved efficacy in patients undergoing hemodialysis for uremia, without an associated increase in adverse events, indicating a favorable safety profile.

### Albumin

**Figure 3:**
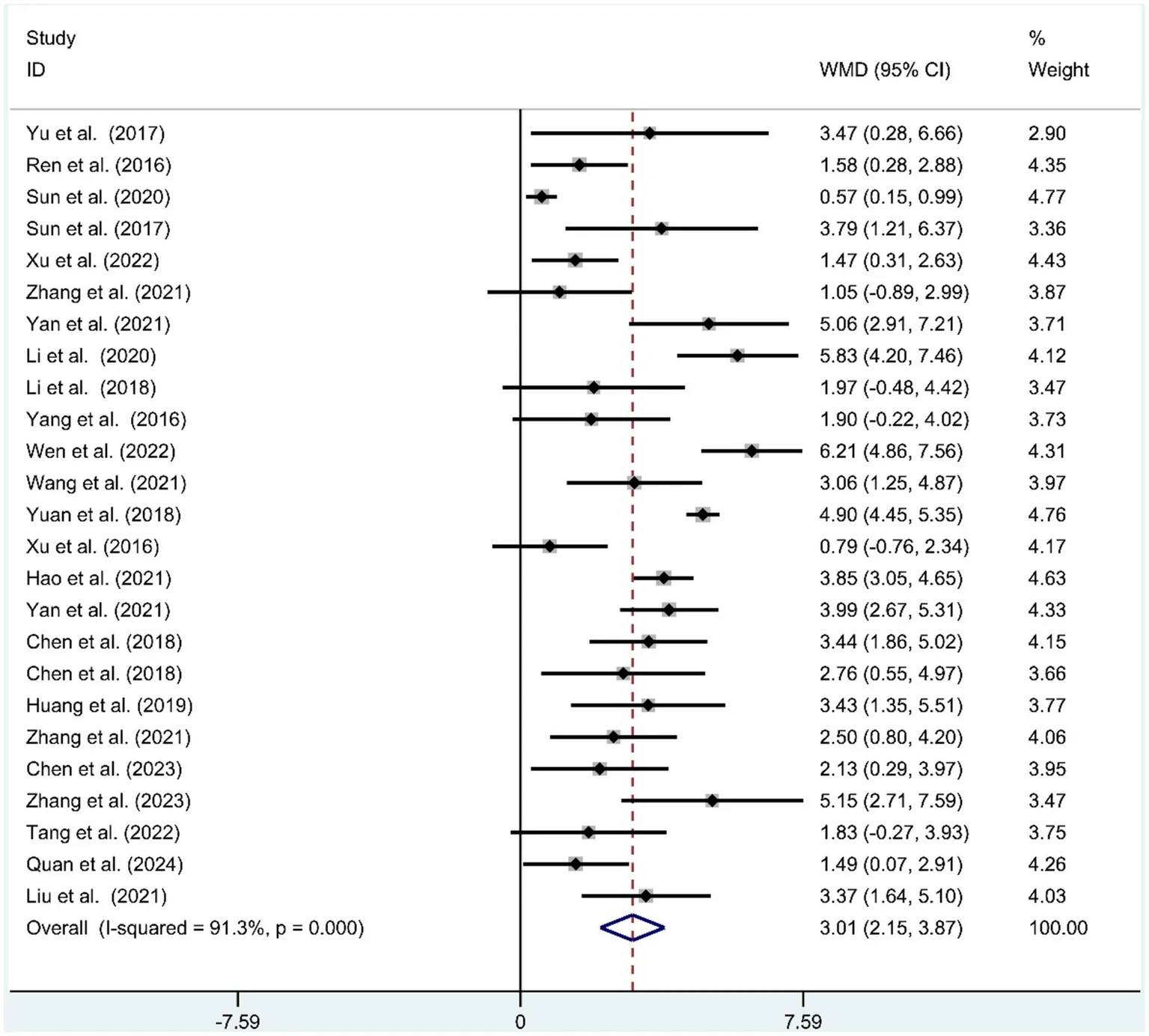
Forest plot illustrating albumin levels.

A total of 25 studies reported data on serum albumin levels. The pooled results demonstrated that the intervention group was associated with a significantly greater increase in albumin levels compared with the control group (WMD = 3.009; 95% CIs: 2.149 to 3.869; *p* < 0.001; Figure 3). Substantial heterogeneity was observed across the included studies (I^2^ = 91.3%; *p* < 0.001; Figure 3). Therefore, a random-effects model was applied.

**Figure 4:**
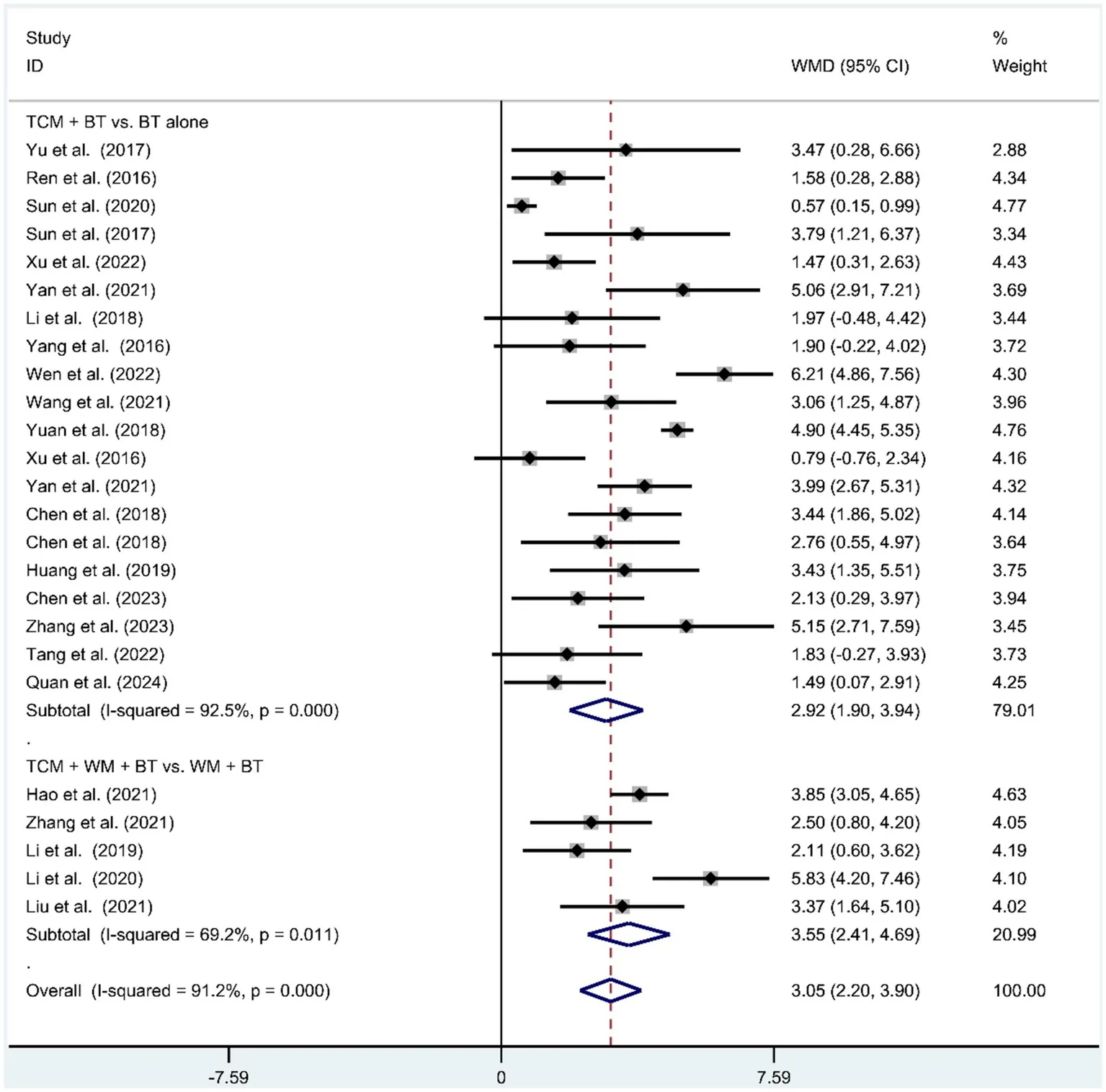
Subgroup analysis of serum albumin levels stratified by different interventions.

For serum albumin, 20 studies compared TCM plus basic therapy (BT) with BT alone. The pooled results showed a significant improvement in albumin levels in the TCM group (WMD = 2.920; 95% CIs: 1.895 to 3.945; *p* < 0.001; Figure 4), with substantial heterogeneity (I^2^ = 92.5%; *p* < 0.001; Figure 4). In the subgroup of five studies comparing TCM plus Western medicine (WM) and BT versus WM plus BT, a similarly significant effect was observed (WMD = 3.549; 95% CIs: 2.408 to 4.689; *p* < 0.001; Figure 4), with moderate heterogeneity (I^2^ = 69.2%; *p* = 0.011; Figure 4).

### Hemoglobin

**Figure 5:**
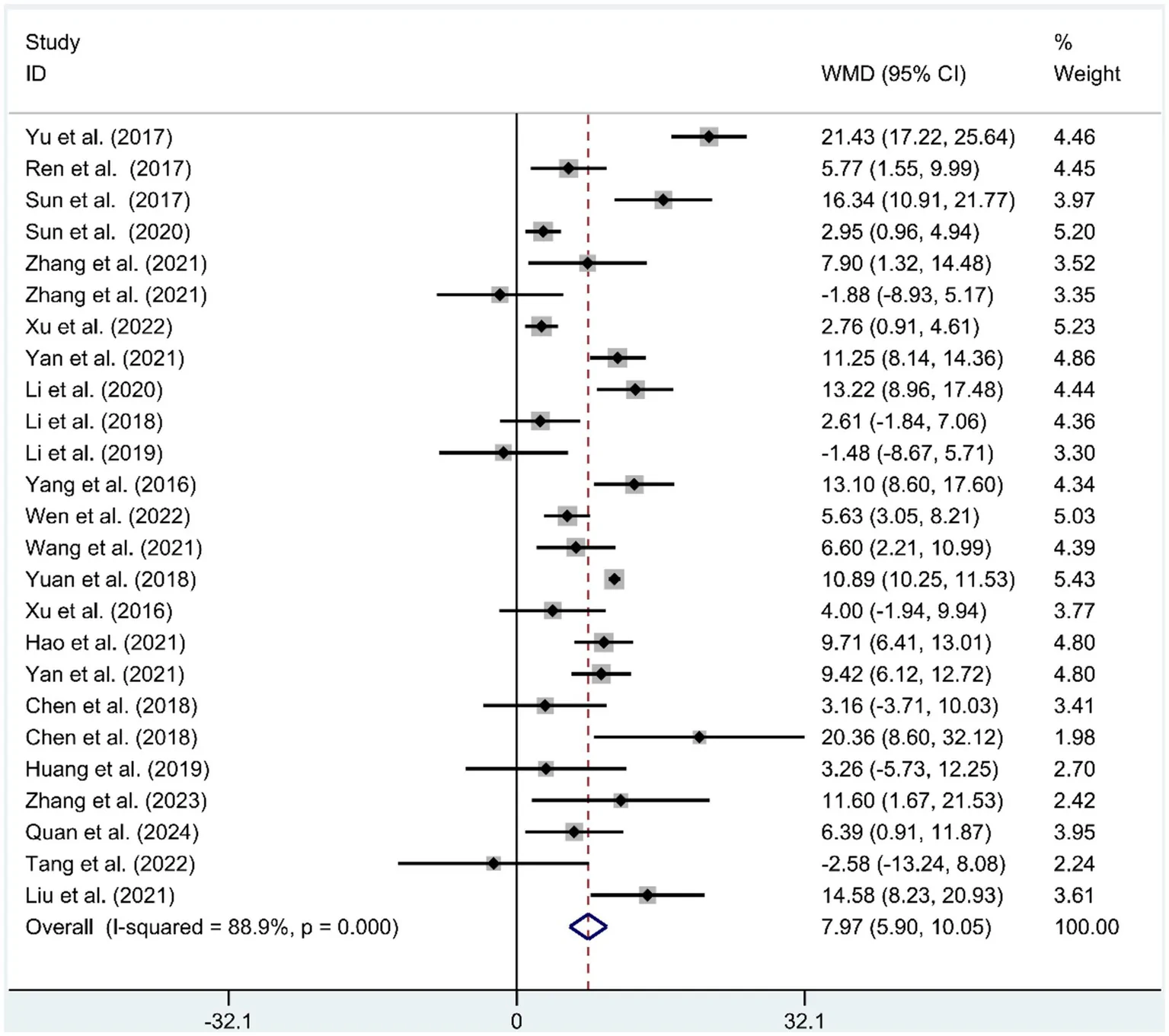
Forest plot illustrating hemoglobin levels.

Hemoglobin levels were reported in a total of 25 investigations. The pooled results demonstrated that the intervention group was associated with a significantly greater increase in hemoglobin levels compared with the control group (WMD = 7.975; 95% CIs: 5.901 to 10.048; *p* < 0.001; Figure 5). Substantial heterogeneity was observed across the included studies (I^2^ = 88.9%, *p* < 0.001; Figure 5), therefore, a random-effects model was applied.

**Figure 6:**
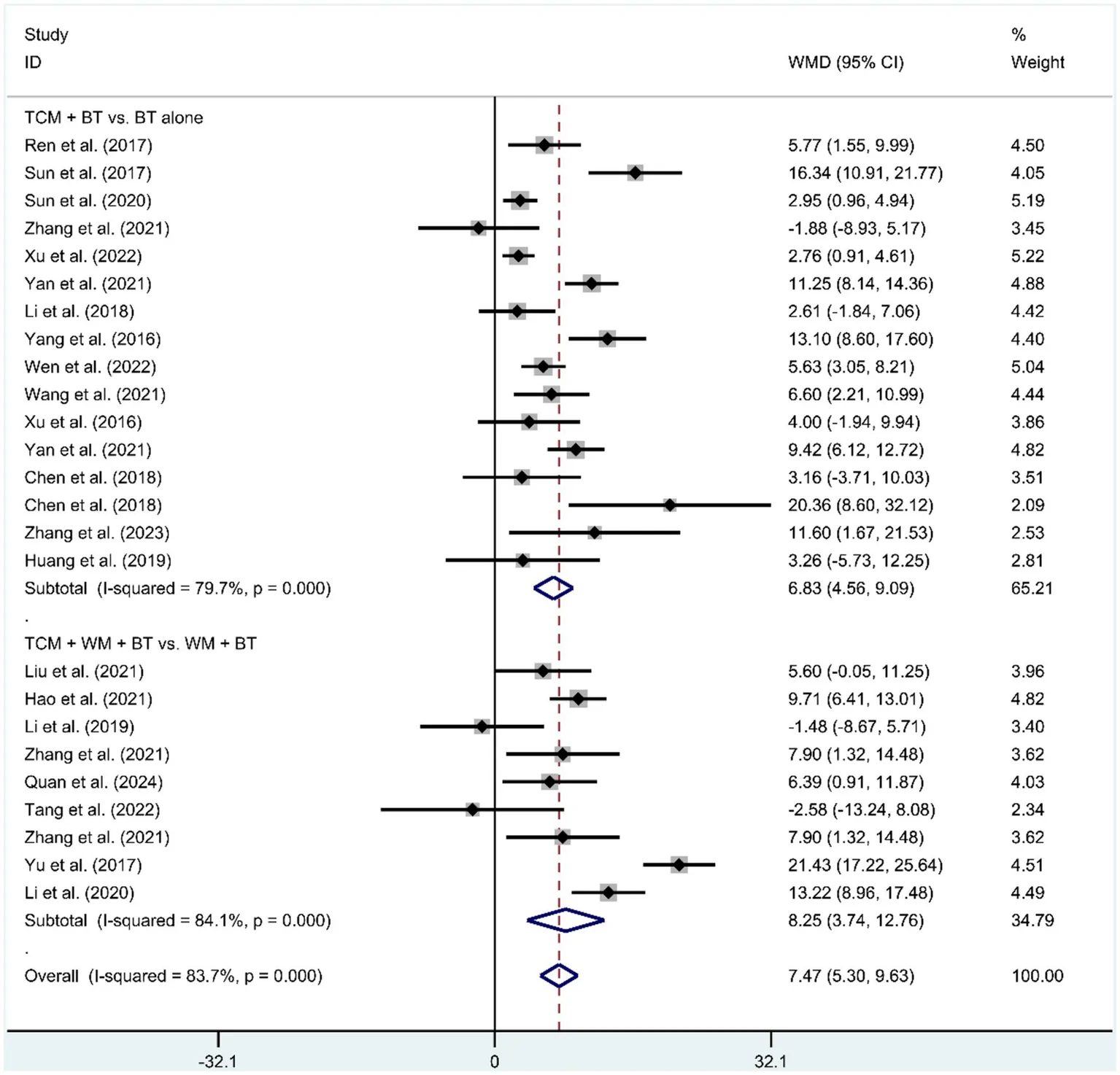
Subgroup analysis of hemoglobin levels stratified by different interventions.

For hemoglobin, 16 studies compared TCM plus BT with BT alone. The pooled results indicated a significant increase in hemoglobin levels in the TCM group (WMD = 6.825; 95% CIs: 4.560 to 9.091; *p* < 0.001; Figure 6), with substantial heterogeneity (I^2^ = 79.7%; *p* < 0.001; Figure 6). In the subgroup of nine studies comparing TCM plus WM and BT versus WM plus BT, a significant effect was also demonstrated (WMD = 8.248, 95% CI: 3.741 to 12.756, *p* < 0.001), with moderate heterogeneity (I^2^ = 84.1%, *p* < 0.001).

### Summary of subgroup findings

Subgroup analyses revealed that traditional Chinese medicine combined with basic therapy, either alone or in addition to Western medicine, was consistently associated with significant improvements in both serum albumin and hemoglobin levels compared with control regimens. Although heterogeneity remained substantial in most subgroups, it was notably reduced for albumin in the TCM + WM + BT versus WM + BT subgroup (I^2^ = 69.2%) compared with the TCM + BT versus BT alone subgroup (I^2^ = 92.5%). However, for hemoglobin, heterogeneity remained high in both subgroups (I^2^ = 79.7% vs. 84.1%), indicating that the addition of Western medicine did not reduce heterogeneity for this outcome. These findings support the potential benefit of TCM as an adjunctive therapy for improving nutritional status in patients undergoing maintenance hemodialysis. The above results are presented in Table 4.

### BMI, TSF and MAC

**Figure 7:**
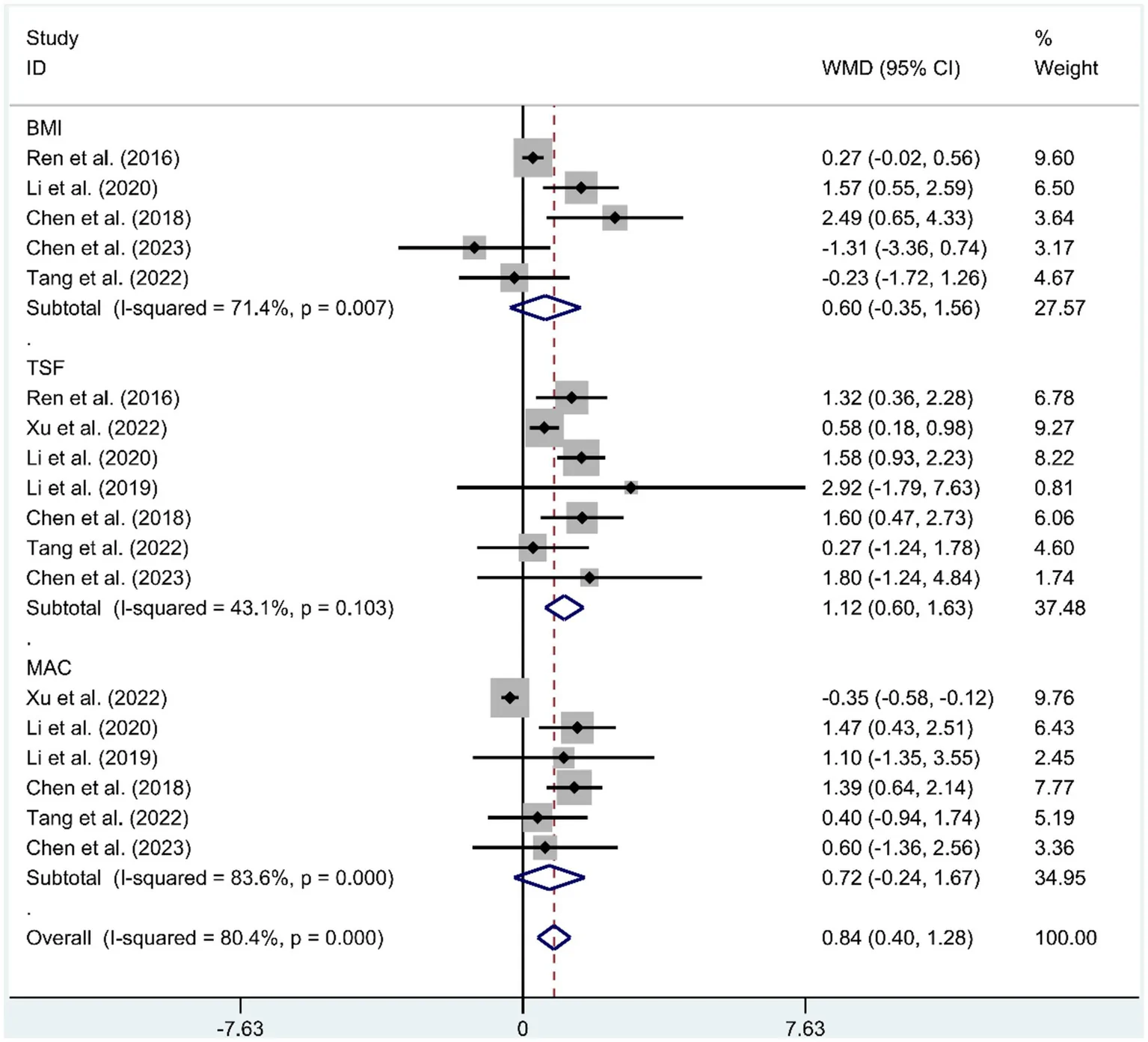
Forest plot illustrating BMI + TSF + MAC.

BMI data were published in five trials. The pooled results showed no statistically significant difference between the intervention group and the control group (WMD = 0.604; 95% CIs: −0.352 to 1.560; *p* = 0.216; Figure 7). Moderate heterogeneity was observed across the studies (I^2^ = 71.4%; *p* = 0.007; Figure 7), therefore, a random-effects model was applied. Data on TSF were provided in seven research. The pooled results demonstrated that the intervention group was associated with a significant increase in TSF compared with the control group (WMD = 1.119; 95% CIs: 0.605 to 1.634; *p* < 0.001; Figure 7). Low heterogeneity was detected among the studies (I^2^ = 43.1%; *p* = 0.103; Figure 7). Data on MAC was published in six research. The pooled results revealed no statistically significant difference between the intervention and control groups (WMD = 0.717; 95% CIs: −0.241 to 1.675; *p* = 0.143; Figure 7). Substantial heterogeneity was observed across the included studies (I^2^ = 83.6%, *p* < 0.001; Figure 7).

### Leptin

**Figure 8:**
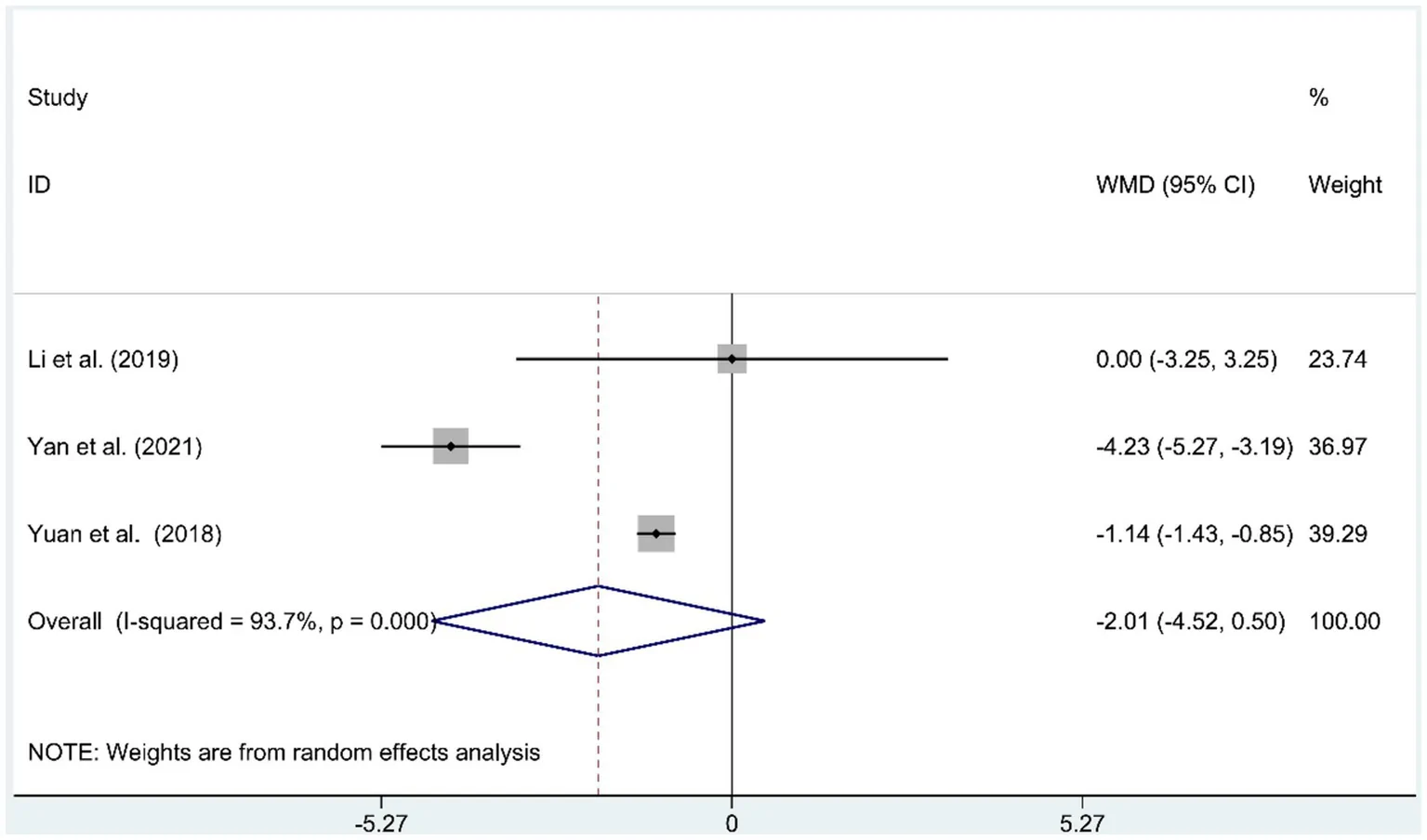
Forest plot illustrating leptin.

Three studies reported data on serum leptin levels. The pooled results showed no statistically significant difference between the intervention group and the control group (WMD = −2.012; 95% CIs: −4.520 to 0.496; *p* = 0.116; Figure 8). Substantial heterogeneity was observed across the included studies (I^2^ = 93.7%; *p* < 0.001; Figure 8), a random-effects model was used.

### Residual kidney function

**Figure 9:**
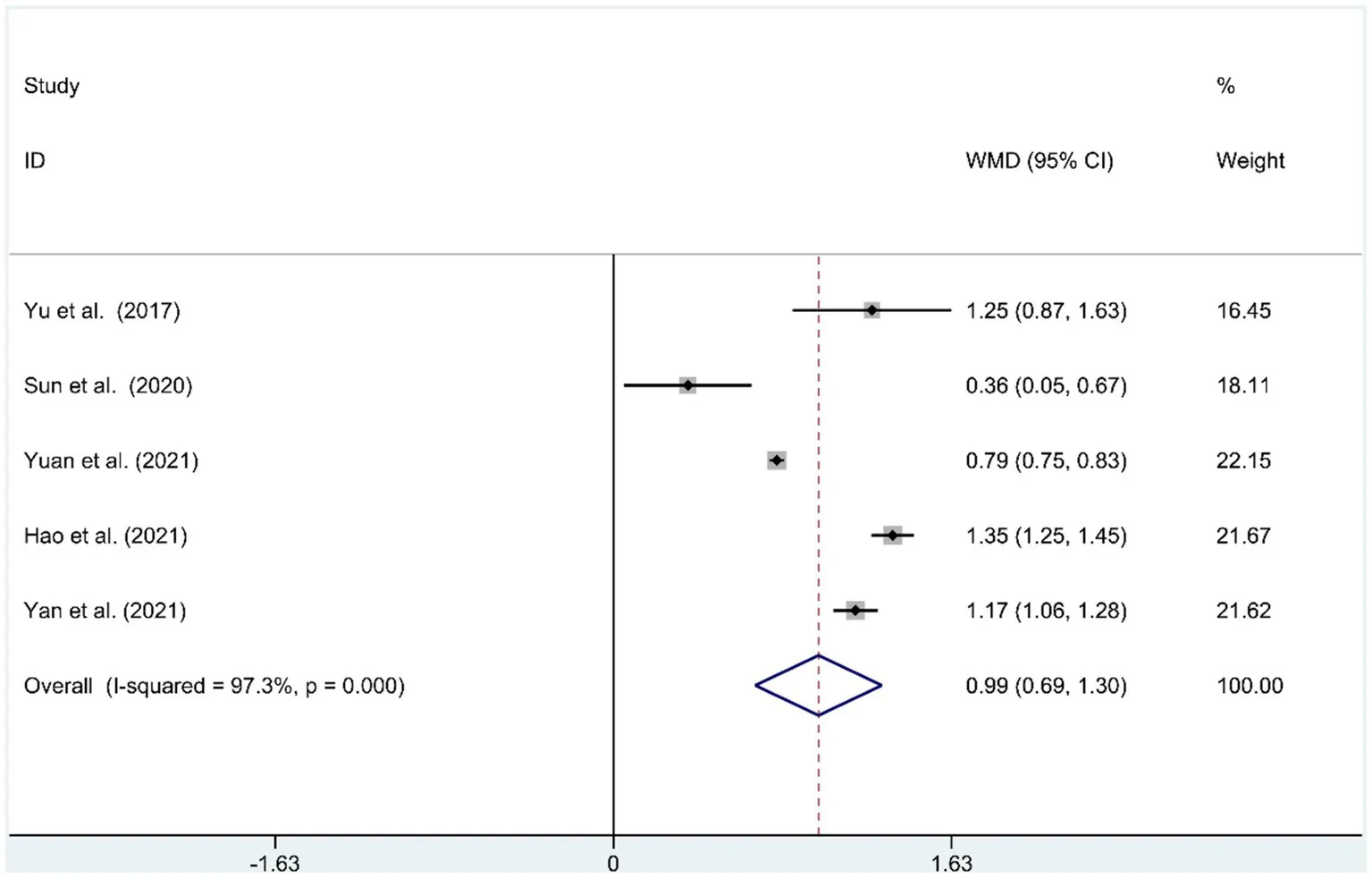
Forest plot illustrating residual kidney function.

Five studies reported data on residual kidney function. The pooled results demonstrated that the intervention group was associated with a significant improvement in residual kidney function compared with the control group (WMD = 0.991; 95% CIs: 0.685 to 1.297; *p* < 0.001; Figure 9). Substantial heterogeneity was observed across the included studies (I^2^ = 97.3%; *p* < 0.001; Figure 9). a random-effects model was used.

### Transferrin

**Figure 10:**
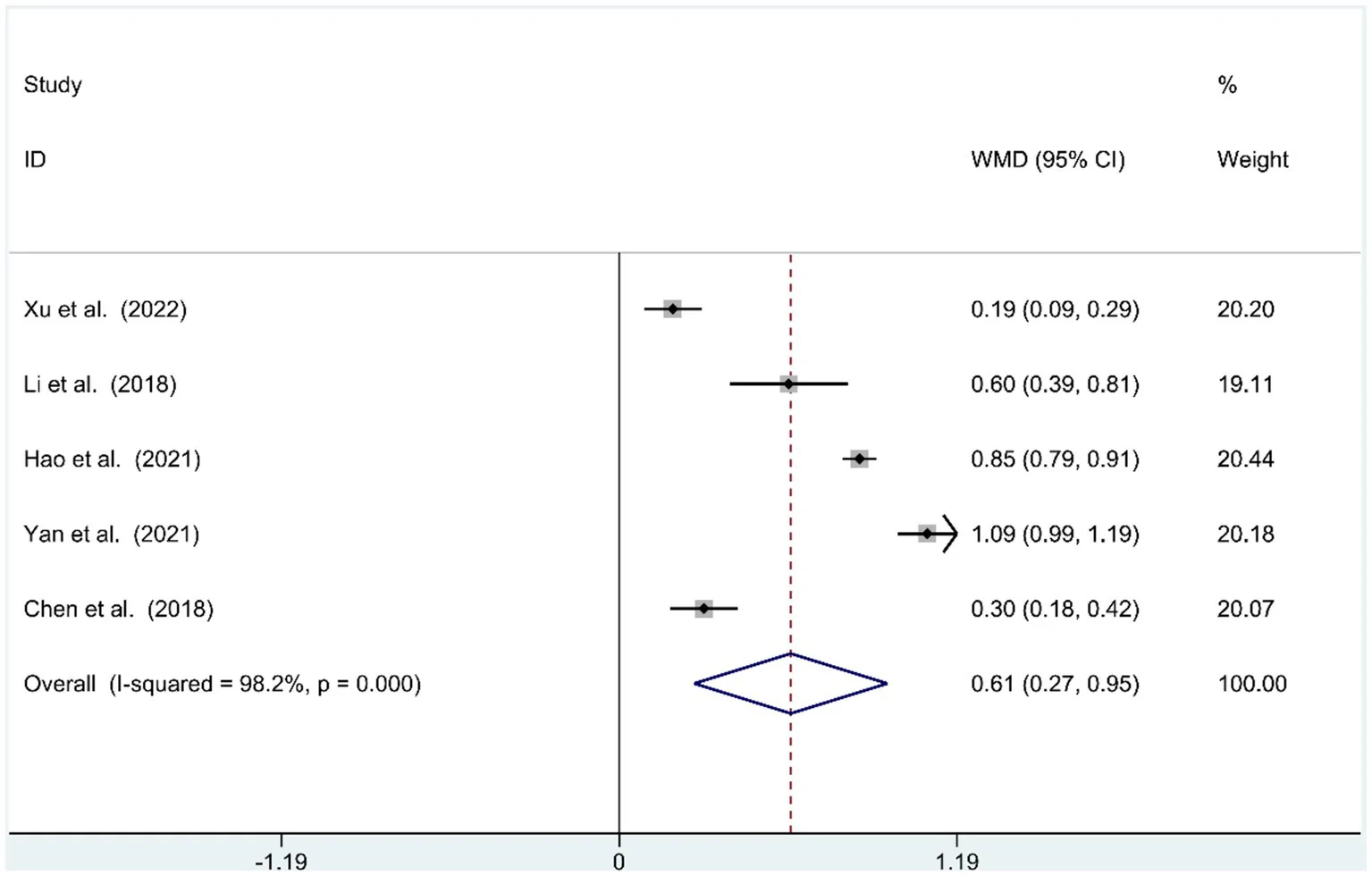
Forest plot illustrating transferrin.

Five studies reported data on transferrin levels. The pooled results showed that the intervention group was associated with a significant increase in transferrin levels compared with the control group (WMD = 0.607; 95% CIs: 0.266 to 0.948; *p* < 0.001; Figure 10). Substantial heterogeneity was detected among the studies (I^2^ = 98.2%; *p* < 0.001; Figure 10), a random-effects model was applied.

### Total cholesterol

**Figure 11:**
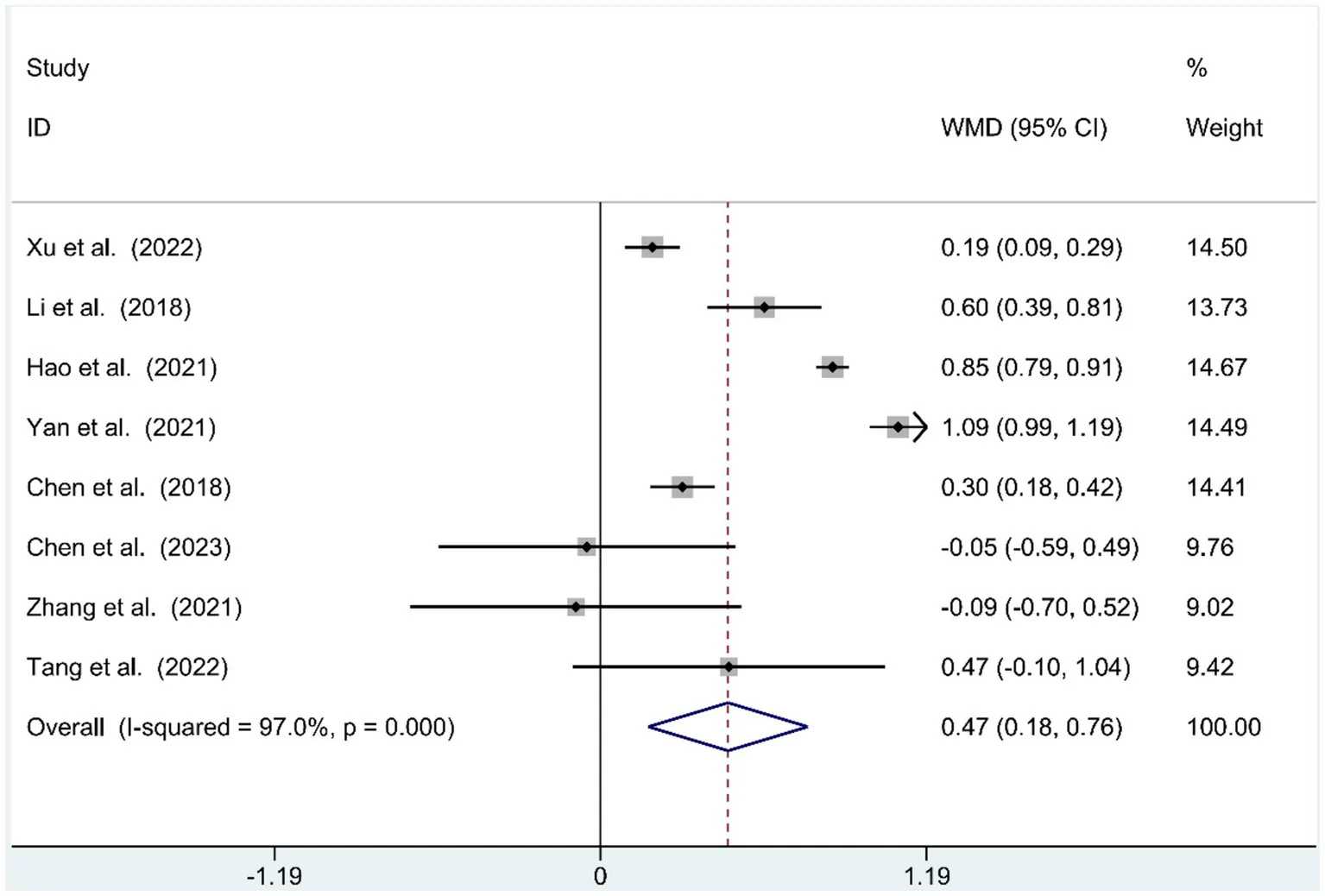
Forest plot illustrating total cholesterol.

Eight studies reported data on total cholesterol levels. The pooled results demonstrated that the intervention group was associated with a significant increase in total cholesterol levels compared with the control group (WMD = 0.467; 95% CIs: 0.175 to 0.759; *p* = 0.002; Figure 11). Substantial heterogeneity was observed across the included studies (I^2^ = 97.0%; *p* < 0.001; Figure 11). Therefore, a random-effects model was used.

### Subjective global assessment

**Figure 12:**
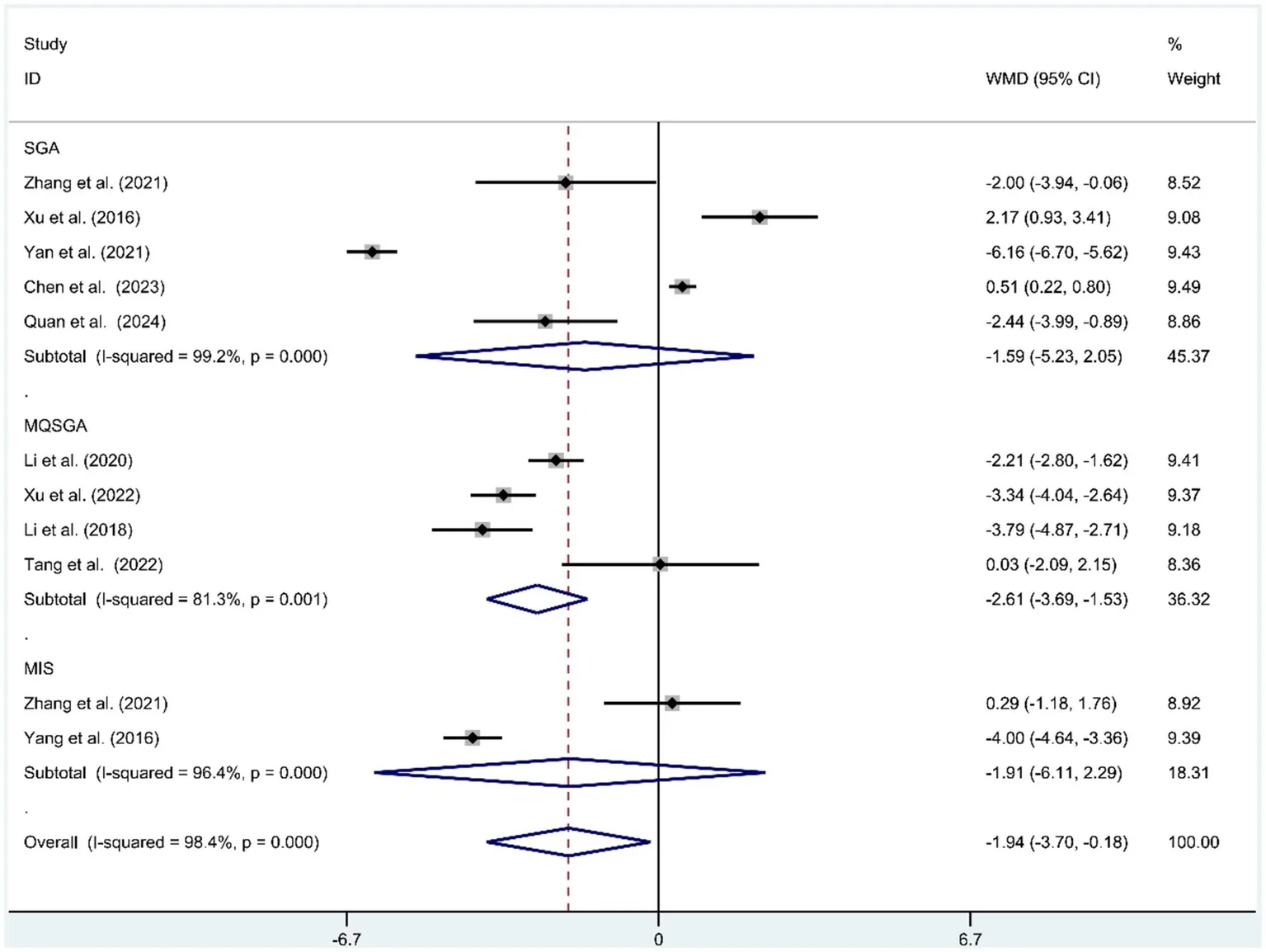
Forest plot of SGA, MQSGA, and MIS scores.

Five studies reported data on SGA scores. The pooled results showed no statistically significant difference between the intervention group and the control group (WMD = −1.587; 95% CI: −5.228 to 2.053; *p* = 0.393; Figure 12). Substantial heterogeneity was detected among the studies (I^2^ = 99.2%; *p* < 0.001; Figure 12), necessitating the use of a random-effects model.

### Modified quantitative subjective global assessment

Four studies reported data on MQSGA scores. The pooled results demonstrated that the intervention group was associated with a significant improvement (lower scores indicating better nutritional status) compared with the control group (WMD = −2.613; 95% CIs: −3.694 to −1.532; *p* < 0.001; Figure 12). Substantial heterogeneity was observed across the included studies (I^2^ = 81.3%; *p* = 0.001; Figure 12), therefore, a random-effects model was applied.

### Malnutrition inflammation score

Two studies reported data on MIS scores. The pooled results revealed no statistically significant difference between the intervention and control groups (WMD = −1.909; 95% CI: −6.111 to 2.294; *p* = 0.373; Figure 12). Substantial heterogeneity was detected among the studies (I^2^ = 96.4%; *p* < 0.001; Figure 12), and a random-effects model was used.

## Discussion

In this meta-analysis of 26 randomized controlled trials including 2,263 maintenance hemodialysis patients, adding traditional Chinese medicine (TCM) to background therapy – with or without conventional Western drugs—was associated with improvements in overall response rate, serum albumin, hemoglobin, transferrin, triceps skinfold thickness, residual kidney function, and MQSGA score. No clear effects were observed on BMI, MAC, leptin, SGA, or MIS. Adding Western medicine to TCM reduced statistical heterogeneity in albumin and hemoglobin outcomes, possibly because Western medicine regimens were more standardized across studies. Adverse event rates did not differ between groups. These findings suggest that TCM may be a safe adjunctive therapy for improving nutritional status in this population.

Although TCM theory attributes malnutrition in hemodialysis patients to spleen-kidney qi deficiency and blood stasis (19, 33); and TCM interventions are often intended to supplement qi and activate blood circulation (37, 38). The direct evidence linking these theoretical constructs to the observed clinical outcomes remains limited. More concretely, some studies have proposed that TCM may act via anti-inflammatory pathways, preservation of residual renal function, regulation of protein metabolism, and modulation of gut microbiota (33, 36), and that combination with Western agents such as L-carnitine or erythropoietin might yield complementary effects (7, 39, 40). These mechanistic hypotheses are partially supported by our meta-analytic findings—namely improvements in albumin, hemoglobin, and residual renal function—but should be interpreted with caution given the heterogeneity across studies and the lack of direct mechanistic measurements in the original trials. Nonetheless, the available data do not suggest an increased risk of adverse events with such combined approaches (13, 23, 27, 33).

Several limitations should be acknowledged. First, all included RCTs were conducted in Chinese populations, and because TCM is used mainly in East Asia and rarely in Europe or the US, our findings may not be directly generalizable to Western settings. Second, the 26 trials used diverse TCM formulas, dosages, and treatment durations without standardized protocols. Long-term safety, hard endpoints (e.g., hospitalization, mortality), and mechanistic insights were not addressed. Furthermore, TCM syndrome scoring is inherently subjective, and the biological pathways underlying nutritional improvements remain unclear. The substantial heterogeneity and methodological weaknesses of the primary studies preclude firm conclusions about effect consistency. Additionally, we acknowledge that grouping various TCM modalities—including herbal decoctions, patent medicines, injections, acupuncture, and auricular therapy—into a single category may have concealed potential differences in their respective effects, and this issue warrants further investigation in future studies. Thus, future research should employ larger samples, double-blind designs, standardized regimens, extended follow-up, and objective outcomes to confirm TCM’s benefits and elucidate its mechanisms.

Despite these limitations, we believe our meta-analysis provides clinically relevant evidence supporting TCM as a potential adjunctive therapy for nutritional management, and synthesizes a decade of data to guide future trial design. We plan to disseminate these findings via peer-reviewed journals, conferences, and clinician-oriented materials, while explicitly noting the population-specific context and the urgent need for validation in diverse populations.

## Conclusion

In summary, malnutrition is common in MHD patients, leading to PEW, frailty, sarcopenia, and other complications, which reduce quality of life and burden families and society. The findings of this meta-analysis suggest that traditional Chinese medicine, as an adjunct to standard care, may be associated with improvements in several nutritional indicators (e.g., serum albumin, hemoglobin) in maintenance hemodialysis patients, without increasing the risk of adverse events. However, owing to substantial heterogeneity across studies and the generally low quality of the included randomized controlled trials, these results should be interpreted with caution. However, given that all included trials were conducted in Chinese populations and that considerable heterogeneity existed across studies, these findings should be interpreted cautiously and require confirmation in future high-quality, multicenter randomized controlled trials with standardized protocols and longer follow-up. Despite these caveats, we believe the current evidence provides a useful reference for clinical decision-making and for guiding the design of future investigations.

## Data Availability

The datasets presented in this study can be found in online repositories. The names of the repository/repositories and accession number(s) can be found in the article/Supplementary material.

## References

[1] Webster AC Nagler EV Morton RL Masson P. Chronic kidney disease. Lancet. (2017) 389:1238–52. doi: 10.1016/S0140-6736(16)32064-5, 27887750

[2] Reid C Seymour J Jones C. A thematic synthesis of the experiences of adults living with hemodialysis. Clin J Am Soc Nephrol. (2016) 11:1206–18. doi: 10.2215/CJN.10561015, 27246010 PMC4934845

[3] Rafferty Q. Global, regional, and national prevalence of kidney failure with replacement therapy and associated aetiologies, 1990–2023: a systematic analysis for the global burden of disease study 2023. Lancet Glob Health. (2025) 13:e1378–95. 40712611 10.1016/S2214-109X(25)00198-6

[4] Morton RL Schlackow I Mihaylova B Staplin ND Gray A Cass A. The impact of social disadvantage in moderate-to-severe chronic kidney disease: an equity-focused systematic review. Nephrol Dial Transplant. (2016) 31:46–56. doi: 10.1093/ndt/gfu394, 25564537

[5] Yi Ren ZZ. A meta-analysis of the efficacy of traditional Chinese medicine on malnutrition state after maintenance hemodialysis in patients with spleen and kidney deficiency. Clin J Chin Med. (2025) 17:114–20.

[6] Carrero JJ Thomas F Nagy K Arogundade F Avesani CM Chan M . Global prevalence of protein-energy wasting in kidney disease: a Meta-analysis of contemporary observational studies from the International Society of Renal Nutrition and Metabolism. J Ren Nutr. (2018) 28:380–92. doi: 10.1053/j.jrn.2018.08.006, 30348259

[7] MacLaughlin HL Friedman AN Ikizler TA. Nutrition in kidney disease: Core curriculum 2022. Am J Kidney Dis. (2022) 79:437–49. doi: 10.1053/j.ajkd.2021.05.024, 34862042

[8] Ueda N Takasawa K. Role of Hepcidin-25 in chronic kidney disease: Anemia and beyond. Curr Med Chem. (2017) 24:1417–52. doi: 10.2174/0929867324666170316120538, 28302014

[9] Yang JLQ Li DY. Effectiveness and safety of roxadustat for renal anemia in dialysis-dependent chronic kidney disease: a meta-analysis. Chin Gen Pract. (2023) 26:704–10.

[10] Ji L. Development and Clinical Application of a Quality of Life Scale with TCM Characteristics for Maintenance Hemodialysis Patients. Beijing: Beijing University of Chinese Medicine (2025).

[11] Xingyue Quan WL Kang N. Clinical observation of Heidihuang powder on anemia and malnutrition in patients on maintenance hemodialysis. Mod J Integr Tradit Chin West Med. (2024) 33:3431–4.

[12] Li K. Effect of Jianpi Huazhuo Tang on nutritional status and quality of life in patients with maintenance hemodialysis. New Chin Med. (2020) 52:74–7.

[13] Huang X. Effects of Shenkang injection combined with hemodialysis on nutritional status in patients with end-stage renal disease. Shenzhen J Integr Tradit Chin West Med. (2019) 29:37–9.

[14] Zuhong Zhang LH Wu B Liu S. Analysis of the curative effect of Shenlingbaizhu powder on malnutrition after dialysis in patients with uremia. China Foreign Med Treat. (2021) 40:5–8.

[15] Jiebin Chen LL Lai W Li C Lü P Wei L. Effects of the therapy of invigorating the spleen and tonifying the kidney, unblocking the Fu-organs and resolving turbidity on malnutrition and microinflammation status in maintenance hemodialysis patients. J Sichuan Tradit Chin Med. (2018) 36:105–8.

[16] Xiumei Yu YG. Clinical study on self-formulated Yishen Xiezhuo decoction combined with hemodialysis in treating uremia and its effect on renal function. J Emerg Tradit Chin Med. (2017) 26:1806–8.

[17] Xu L. Clinical Study on the Method of Tonifying the Kidney, Invigorating the Spleen, and Enriching Blood for Treating Malnutrition in Maintenance Hemodialysis Patients. Henan: Henan University of Chinese Medicine (2016).

[18] Yang T. The Effects of Uremic Clearance Granule on the Microinflammation and Malnutrition in Maintenance Hemodialysis Patients. Nanchang: Nanchang University (2016).

[19] Ren Y. Clinical Observation on Zusanli Point Injection of Astragalus Injection for Malnutrition in Maintenance Hemodialysis Patients. Henan: Henan University of Chinese Medicine (2016).

[20] Hui Zhang JL Tong Y Cai H. Clinical study of acupoint injection of astragalus injection on hypotension and protein-energy malnutrition in maintenance hemodialysis patients. J Clin Nephrol. (2023) 23:988–94.

[21] Lin Chen SW Qiu M Luan J Yan E Liu P Wang Y . Therapeutic observation of acupoint application for malnutrition in patients on maintenance hemodialysis. Shanghai J Acupunct Moxibustion. (2023) 42:1151–6.

[22] Sun H. Clinical Study on Heluo Ningfeng Granule in Treating Calcium-Phosphorus Metabolism Disorder in Chronic Renal Failure. Qingdao: Qingdao University (2017).

[23] Chen J Lou J Yang J. Effects of Shenkang injection combined with hemodialysis on nutritional status in patients with end-stage renal disease. Tianjin J Tradit Chin Med. (2018) 35:816–8.

[24] Li J Zhao W Zhou Y. Effects of Xiangsha Yangwei pills combined with moxibustion on the spleen and stomach on malnutrition in uremia patients with spleen deficiency. Henan Tradit Chin Med. (2018) 38:1403–5.

[25] Yuan Z Zou L. Efficacy of dampness-resolving and turbidity-lowering Chinese herbs as an adjunctive therapy to hemodialysis in treating chronic renal failure and its influence on nutritional status and gastrointestinal hormone levels. Mod J Integr Tradit Chin West Med. (2018) 27:245–248+252.

[26] Li Y. Treating Malnutrition in Maintenance Hemodialysis Patients with Harmonizing Method: A Prospective, Single-Center, Randomized Controlled Clinical Study. Beijing: Beijing University of Chinese Medicine (2019).

[27] Sun X. Clinical Research on Black Dihuang Pill Protecting Residual renal Function in Maintenance Hemodialysis Patients. Shandong: Shandong University of Traditional Chinese Medicine (2020).

[28] Wang W. Clinical study on the effect of Qizhu capsule on microinflammatory state and Th1/Th2 in maintenance hemodialysis patients with deficiency of spleen and kidney and interweavement of dampness and stagnation of blood. J Shanxi Univ Chin Med. (2021)

[29] Yan Z Liu Z. Effect of Shenqi Qingdu decoction on residual renal function and nutritional status of patients with maintenance hemodialysis. Clin J Tradit Chin Med. (2021) 33:1533–6.

[30] Yan Z Shen J Hu F. Analysis of clinical curative effect of Shenshuai recipe on patients with maintenance hemodialysis. Chin Arch Tradit Chin Med. (2021) 39:14–7.

[31] Zhang P. Clinical Observation on Five-Color Peiyuan Guben Ointment in Improving Protein-Energy Wasting in Maintenance Hemodialysis Patients. China: Hunan University of Chinese Medicine (2021).

[32] Liu T Lv Q Chen X Zhang W Xiong W. Effect of Runfei Yishen decoction combined with levocarnitine on malnutrition, micro - inflammatory status and quality of life in patients with uremia treated with maintenance hemodialysis. Mod J Integr Tradit Chin West Med. (2021) 30:364–8.

[33] Hao J. Effects of modified Taohong Siwu decoction combined with levocarnitine on nutritional status in maintenance hemodialysis patients. Shaanxi J Tradit Chin Med. (2021) 42:903–6.

[34] Wen L. Clinical observation on Shenkang injection combined with hemodialysis in treating chronic renal failure. Guangming J Chin Med. (2022) 37:3767–9.

[35] Xu B Wen J Wu L Chen J. Effect of Jianpi Huazhuo ointment on protein-energy wasting in hemodialysis patients based on amino acid and carnitine metabolism. Med J West China. (2022) 34:1635–40.

[36] Tang A. Clinical effect of Jianpi Xiaozhi Ointment on Protein Energy Wasting in Patients with Maintenance Hemodialysis(Spleen and Kidney qi Deficiency). Chengdu: Chengdu University of Traditional Chinese Medicine (2022).

[37] Chen J Peng H Yuan Z Zhang K Xiao L Huang J . Combination with anthropometric measurements and MQSGA to assess nutritional status in Chinese hemodialysis population. Int J Med Sci. (2013) 10:974–80. doi: 10.7150/ijms.5811, 23801883 PMC3691795

[38] Ding L. Nutritional status assessment and clinical significance in non-dialysis chronic kidney disease patients. Assessment and Analysis of Nutritional Status in non-dialytic Chronic Kidney Disease Patients. (2017) 15:31–2.

[39] Yan EP Li XJ Wang SH Luan J Liu P Ke YS . Study on the characteristics of traditional Chinese medicine syndromes based on statistical analysis methods in hemodialysis patients of end-stage renal disease. China J Tradit Chin Med Pharm. (2025) 40:3851–5.

[40] Nephrology Branch, C.M.D.A.E.P.f.N.G., Committee of Kidney Diseases, Chinese Society of Integrated Traditional and Western Medicine. Chinese clinical practice guideline for nutritional management of chronic kidney disease (2021). Natl Med J China. (2021) 101:539–59.

