# Supplementary material for: Efficacy of traditional Chinese medicine in improving nutritional status among Chinese maintenance hemodialysis patients: a meta-analysis of the last decade
Source: Front Nutr. 2026 Aug 13;13:1852062. doi: 10.3389/fnut.2026.1852062 (PMC13520736; doi:10.3389/fnut.2026.1852062)
Supplement: Supplementary file 1 [file Data_Sheet_1.PDF]

Supplementary File. Complete search strings for each database (March 2016 – March 2026)

**Database**	**Search string**
**PubMed**	("Renal Dialysis"[MeSH] OR "Hemodialysis"[Title/Abstract] OR "Extracorporeal Dialysis"[Title/Abstract] OR "Maintenance Hemodialysis"[Title/Abstract]) AND ("Nutritional Status"[MeSH] OR "Malnutrition"[MeSH] OR "Nutritional Deficiency"[Title/Abstract] OR "Undernutrition"[Title/Abstract]) AND ("Medicine, Chinese Traditional"[MeSH] OR "Traditional Chinese Medicine"[Title/Abstract] OR "Chinese Herbal Medicine"[Title/Abstract]) AND ("Randomized Controlled Trial"[Publication Type] OR "randomized"[Title/Abstract] OR "placebo"[Title/Abstract])
**Embase**	('renal dialysis'/exp OR 'hemodialysis':ab,ti OR 'extracorporeal dialysis':ab,ti) AND ('nutritional status'/exp OR 'malnutrition'/exp OR 'undernutrition':ab,ti) AND ('traditional chinese medicine'/exp OR 'chinese herbal medicine':ab,ti) AND ('randomized controlled trial'/exp OR 'randomized':ab,ti OR 'placebo': ab,ti)
**Web of Science**	TS= ("renal dialysis" OR "hemodialysis" OR "extracorporeal dialysis" OR "maintenance hemodialysis") AND TS= ("nutritional status" OR "malnutrition" OR "nutritional deficiency" OR "undernutrition") AND TS= ("traditional chinese medicine" OR "chinese herbal medicine" OR "tcm") AND TS= ("randomized controlled trial" OR "randomized" OR "placebo")

**CNKI (Chinese)**	(SU='血液透析' OR SU='维持性血液透析' OR SU='连续性血液净化' OR SU='血透' OR SU='持续血液净化') AND (SU='营养状况' OR SU='营养不良' OR SU='营养状态' OR SU='营养') AND (SU='中药' OR SU='中医' OR SU='中医药') AND (FT='随机' OR FT='随机对照' OR FT='RCT')
**Wanfang Data**	题名或关键词:(血液透析 OR 维持性血液透析 OR 血透 OR 连续性血液净化) 并且 题名或关键词:(营养状况 OR 营养不良 OR 营养状态) 并且 题名或关键词:(中药 OR 中医 OR 中医药) 并且 全文:(随机 OR 随机对照 OR RCT)
**CQVIP (VIP)**	(U='血液透析' OR U='维持性血液透析' OR U='血透') AND (U='营养状况' OR U='营养不良') AND (U='中药' OR U='中医') AND (FT='随机')
**ClinicalTrials.gov**	("hemodialysis" OR "renal dialysis" OR "maintenance hemodialysis") AND ("nutritional status" OR "malnutrition") AND ("traditional chinese medicine" OR "chinese herbal medicine")

Notes: The search period was from March 1, 2016 to March 31, 2026; Language was restricted to Chinese or English; MeSH terms (PubMed) and Emtree terms (Embase) were used where applicable, combined with free-text words (title/abstract); For Chinese databases, subject fields (SU/U) and full-text (FT) were used as appropriate; No additional filters (e.g., study design) were applied beyond those shown.
